# Proximal Detection of Traces of Energetic Materials with an Eye-Safe UV Raman Prototype Developed for Civil Applications

**DOI:** 10.3390/s16010008

**Published:** 2015-12-22

**Authors:** Roberto Chirico, Salvatore Almaviva, Francesco Colao, Luca Fiorani, Marcello Nuvoli, Wenka Schweikert, Frank Schnürer, Luigi Cassioli, Silvana Grossi, Daniele Murra, Ivano Menicucci, Federico Angelini, Antonio Palucci

**Affiliations:** 1 ENEA, FSN-TECFIS-DIM, Via E. Fermi 45, Frascati (Rome) 00044, Italy; salvatore.almaviva@enea.it (S.A.); francesco.colao@enea.it (F.C.); luca.fiorani@enea.it (L.F.); marcello.nuvoli@enea.it (M.N.); ivano.menicucci@enea.it (I.M.); federico.angelini@enea.it (F.A.); antonio.palucci@enea.it (A.P.); 2 Fraunhofer Institute for Chemical Technology ICT, Joseph-von-Fraunhofer-Strasse 7, Pfinztal 76327, Germany; wenka.schweikert@ict.fraunhofer.de (W.S.); frank.schnuerer@ict.fraunhofer.de (F.S.); 3 Italian Air Force, Comando Logistico, 1^Divisione, Centro Sperimentale Volo, Reparto Armamento, Via di Pratica di Mare 45, Pomezia (Rome) 00040, Italy; luigi.cassioli@aeronautica.difesa.it (L.C.); silvana.grossi@aeronautica.difesa.it (S.G.); 4 ENEA, FSN-FUSPHY-SAD, Via E. Fermi 45, Frascati (Rome) 00044, Italy; daniele.murra@enea.it

**Keywords:** explosives, Raman Spectroscopy, proximal detection, laser

## Abstract

A new Raman-based apparatus for proximal detection of energetic materials on people, was developed and tested for the first time. All the optical and optoelectronics components of the apparatus, as well as their optical matching, were carefully chosen and designed to respect international eye-safety regulations. In this way, the apparatus is suitable for civil applications on people in public areas such as airports and metro or railway stations. The acquisition software performs the data analysis in real-time to provide a fast response to the operator. Moreover, it allows for deployment of the apparatus either as a stand alone device or as part of a more sophisticated warning system architecture made up of several sensors. Using polyamide as substrate, the apparatus was able to detect surface densities of ammonium nitrate (AN), 2-methyl-1,3,5-trinitrobenzene (TNT), 3-nitrooxy-2,2-bis(nitrooxymethyl)propyl] nitrate (PETN) and urea nitrate (UN) in the range of 100–1000 μg/cm^2^ at a distance of 6.4 m using each time a single laser pulse of 3 mJ/cm^2^. The limit of detection calculated for AN is 289 μg/cm^2^. AN and UN provided the highest percentages of true positives (>82% for surface densities of 100–400 μg/cm^2^ and fingerprints) followed by TNT and PETN (17%–70% for surface densities of 400–1000 μg/cm^2^ and fingerprints).

## 1. Introduction

In the last decades there have been several terrorist attacks in different cities, which have raised the need for new, reliable and effective instrumentation for the detection of explosives and their precursors at trace levels for homeland security applications. In particular, attacks against buses, trains, subways, *etc.*, is a relatively recent phenomenon [1]. Since 1970, transportation has been an increasingly attractive target for terrorists [2]. Places where large crowds congregate are prime targets of opportunity for suicide bomber attacks (Madrid, 2004; London, 2005; Mumbai, 2006; Moscow, 2010; Volgograd, 2013). A suicide attack is usually performed with an improvised explosive device (IED), which can come in many forms, ranging from a small pipe bomb to a sophisticated device. Many commonly available materials, such as fertilizer, gunpowder and hydrogen peroxide, can be used to prepare an IED [3].

Raman-based technologies are potential tools for the detection of explosives at a certain distance due to recent technical improvements [4]. The instantaneous inelastic scattering of incident photons by target molecules produces Raman spectra that uniquely identify chemical substances. The challenging requirements to deal with in the development of Raman technologies is to achieve a high detection sensitivity for trace explosive materials at a certain distance and also a high selectivity for a reliable identification of the substance on an interfering background.

Most solid explosives have low vapor pressures at room temperature (parts per trillion (ppt) to parts per million (ppm)), and military grade explosives can be detected on persons who have handled explosives even 48 h after exposure [5]. Fingerprints of contaminated individuals can contain some μg of energetic material [6,7].

Several Raman apparatus for the detection of energetic materials, either at trace levels or for quantities in the order of mg, have already been developed by different research groups [8,9]. For instance, Carter *et al.* [10,11] studied the identification of 4%–8% solid explosives in dry sand at a distance up to 50 m utilizing the 532-nm output from a Nd:YAG laser. Gaft and Nagli [12] designed and tested a Raman system for detecting high explosive microparticles at up to a 30 m distance in ambient light conditions by using a pulsed Nd:YAG laser at 532 nm and 266 nm. UV-excited Raman signals (266 nm) were 100–200 times stronger than those generated with green excitation (532 nm), which made the trace detection of energetic materials possible. Explosives showed increased molecular Raman cross-sections in the deep UV range [13]. Jander and Noll [14] demonstrated that UV-laser excitations (266 nm) of a target at short distance (10 cm) allow the identification of traces of TNT and ANFO (55 μg/cm^2^). Petterson *et al.* [15] used a Nd:YAG-laser-based instrumentation, operating at 532 nm, for the detection of a number of explosives and precursors in different containers up to a 55 m distance in an outdoor environment. In that study, it was concluded that the detection of explosives is problematic when the 532-nm wavelength is used because there is interference from fluorescence. A hybrid sensor was developed by Moros *et al.* [16] for simultaneous Raman and Laser Induced Breakdown Spectroscopy (LIBS) stand-off measurements of explosives. A Raman multispectral imaging apparatus (532-nm wavelength excitation) was presented by Östmark *et al.* [17] for the stand-off detection of single explosive particles.

To our knowledge, the Raman apparatus already developed can’t be used for civil applications, because the regulations concerning the maximum permissible exposure (MPE) of the cornea and skin to a collimated laser beam were not taken in consideration.

A new Raman-based instrument was developed at the Diagnostic and Metrology Laboratory (UTAPRAD-DIM) of the Italian National Agency for New Technologies, Energy and Sustainable Economic Development (ENEA) for the proximal detection of explosives in public infrastructures. The definition of the detection distance is based on the classification made by the North Atlantic Treaty Organization (NATO): point (less than 10 cm away), proximal (10 cm–200 m), and stand-off (further than 200 m away) ([18] and references therein).

The RAman Detection of EXplosives (RADEX) apparatus was built taking in consideration the constraint of the maximum permissible laser exposure of the human cornea and skin for 8 h. RADEX was developed in the context of the NATO Science for Peace and Security Program STANdoff Detection of EXplosives (STANDEX) project and it was part of an explosive warning system that included fusion of explosive detection sensors designed to work in a mass transit infrastructure such as a metro station. The STANDEX program also included an adaptation and a validation phase of the developed system in real conditions of use (the Big City Trials project, or BCT, 2013). The RADEX apparatus will be presented in this work together with the results obtained during laboratory tests and trials in a metro station, where the instrument was tested in conditions as much as possible close to reality using mock passengers.

## 2. Experimental

### Trace, Fingerprint and Bulk Samples of Energetic Materials

**Figure 1:**
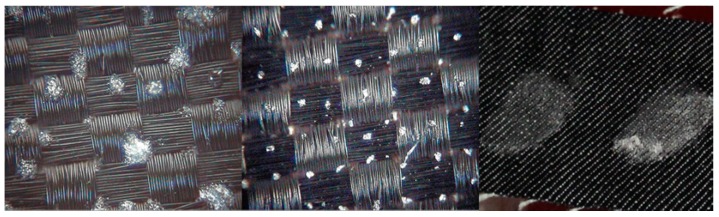
Ammonium nitrate (**left**, 400 μg/cm^2^), urea nitrate (**center**, 400 μg/cm^2^) deposited on synthetic fabrics with the piezoelectric Nano-Plotter™ and fingerprints of TNT on denim (**right**).

The Fraunhofer Institute for Chemical Technology (ICT) contributed to this work by providing traces and fingerprints of energetic materials on fabrics to test the RADEX prototype. A piezoelectric Nano-Plotter™ (PNP, GeSIM, Grosserkmannsdorf, Germany), which can deliver a precise and uniform number of droplets on a surface to produce samples with a broad range of surface concentrations (down to few μg/cm^2^), was used to prepare the trace samples. Fingerprint samples were prepared using a finger made of rubber that simulated a real finger (Figure 1). Future studies should be performed taking in consideration test materials that include the oily substances that are present in fingerprints (e.g., sebum).

Bulk pure explosives, to acquire the Raman spectra for the library of the acquisition software, were provided by the Italian Air Force. Some mg of each explosive were placed in an aluminium holder and irradiated with several laser pulses (DPSS laser, 3 mJ/cm^2^, 266 nm, 10 ns pulse length). The Raman signal was collected and focused with a set of lenses onto the circular-to-slit fiber bundle connected to the spectrograph. A 266-nm ultrasteep long-pass edge filter was used in front of the fiber to reject the Rayleigh radiation. The effect of the energy from several laser pulses on explosives was investigated acquiring a Raman spectrum for each pulse. The laser intensity was sufficiently low that the potential photodegradation or thermal degradation of the explosive samples were not clearly observed. The average peak area of the most intense and representative Raman bands where included in the signal fluctuations between subsequent shots with a relative standard deviation (RSD) below 5% for a total of 30 laser pulses.

The substances object of this study were: 1-nitroguanidine , octahydro-1,3,5,7-tetranitro-1,3,5,7-tetrazocine (HMX), 1,3,5-trinitroperhydro-1,3,5-triazine (RDX), nitrocellulose, [3-nitrooxy-2,2-bis(nitrooxymethyl)propyl] nitrate (PETN), *N*-methyl-*N*,2,4,6-tetranitroaniline (Tetryl), 2-methyl-1,3,5-trinitrobenzene (TNT), 2,4,6-trinitrophenol (picric acid), 2,4,6-trinitrobenzene-1,3-diol (styphnic acid), potassium chlorate (KClO_3_), ammonium nitrate (AN), AN fertilizer, ammonium perchlorate (NH_4_ClO_4_), urea nitrate (UN), sodium azide, 3,4,8,9,12,13-hexaoxa-1,6-diazabicyclo[4.4.4]tetradecane (HMTD), 3,3,6,6,9,9-hexamethyl-1,2,4,5,7,8-hexaoxacyclononane (TATP), home-made TATP.

## 3. Results and Discussion

### 3.1. The RADEX Prototype

**Figure 2:**
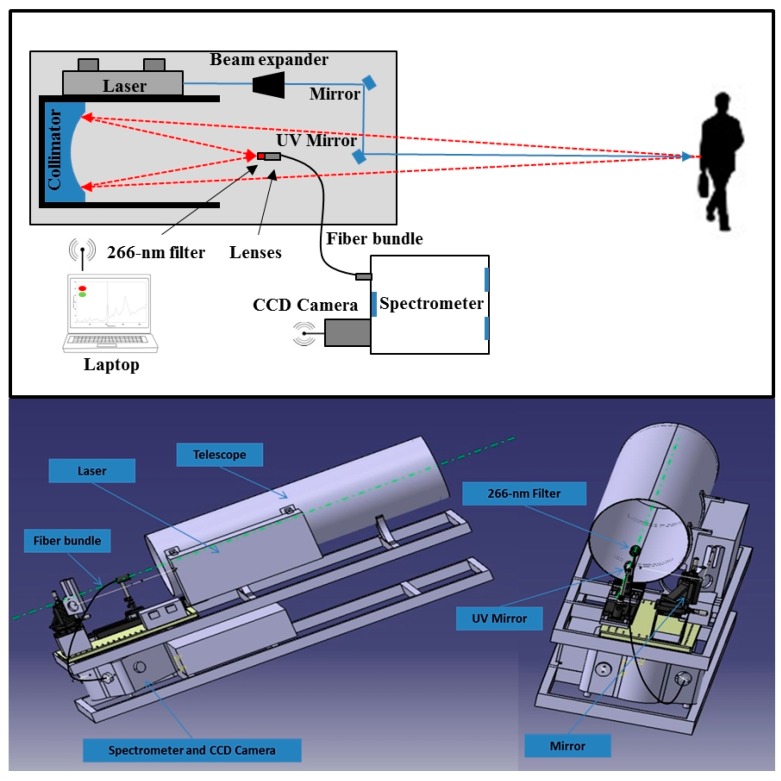
The schematic representation (**above**) and the 3-D model (**below**) of the RADEX system. A single eye-safe laser beam is sent to a target (cloths, backpacks) then, the Raman scattered radiation emitted by the target is collected by a telescope and imaged into the entrance of a Raman spectrometer. At the end, the apparatus provides a spectrum suitable for the explosive detection and identification.

The objective of the RADEX project was the development of a prototype of an eye-safe system for proximal detection of explosive residues on contaminated surfaces for civil applications. The operational principle of RADEX contemplates an eye-safe laser beam that is sent to a target then, the Raman scattered radiation emitted by the target is collected by a telescope and imaged into the entrance of a Raman spectrometer. The following analysis of the radiation provides a spectrum suitable for the explosive detection and identification (Figure 2).

The RADEX system was designed to operate with a single laser pulse (10 ns pulse length) at 266-nm wavelength with a radiant exposure of 3 mJ/cm^2^ provided by a Diode-Pumped Solid-State (DPSS) laser (ATC Semiconductor Devices, St-Petersburg, Russia) equipped with 16 laser diode bars, a Nd:YAG active crystal, and frequency doubling crystals. A special design of the laser cavity provide in first approximation a flat top shape for the laser spot for an homogeneous illumination of the target and to guarantee the respect of the international regulations related to laser safety [19].

The following benefits are encountered working with a 266-nm wavelength: a higher eye-safe energy can be provided, enhanced Raman signals, reduced wavelength overlap of the Raman and fluorescence spectra, the Hartley absorption band of ozone blocks the solar radiation at these wavelengths [19,20].

A beam expander (1.0×–8.0× with adjustable divergence, Sill Optics, Wendelstein, Germany) was located in front of the laser source to have a laser spot at the target distance with a diameter of 1 cm^2^. Raman scattered radiation is collected by the 1-mirror Newton (paraboloid) collimator (focal length = 1 m, diameter = 315 mm, dielectric coating with reflectance >98% from 266 to 300 nm, Optical Surfaces Ltd., England, UK) and it is concentrated in its focal point. The focused light by the telescope is transmitted to the grating of a spectrometer (aperture = f/4.1, 1800 grooves/mm, iHR-320, Horiba Jobin Yvon, Kyoto, Japan) using a 0.75-m long circular (d = 1.95 mm) to slit (0.23 mm × 13.2 mm) anti-reflection coated fiber bundle comprising 53 fibers with a core diameter of 200 μm and a numerical aperture of 0.12 (Leoni, Däniken, Switzerland). A 266-nm ultrasteep long-pass edge filter is used in front of the fiber to reject the strong Rayleigh scattering radiation (average transmittance >90% for 272–600 nm; optical density >6 for 266 nm, RazorEdge^®^, Semrock Inc., Rochester, NY, USA). An iKon-M934 camera (Andor, Belfast, UK) is used as detector (quantum efficiency = 65%, 1024 × 1024 active pixels, sensor size = (13.3 × 13.3) mm, pixel size = (13 × 13) μm, read noise = 6.6 e^−^) and it was run with the following optimal settings: −70 °C, 0.00108 s for the exposure time, full vertical binning, 1 MHz for the pixel readout rate, 4 for the pre-amplifier gain. It was observed that from −40 °C to lower cooling temperatures of the camera, there was no improvements in the signal to noise ratio (SNR) of the Teflon Raman bands. The optical matching between the laser excitation area and the collection/dispersion optical elements was carefully designed with the support of a ray tracing software (Zemax, LLC, Redmond, WA, USA).

**Figure 3:**
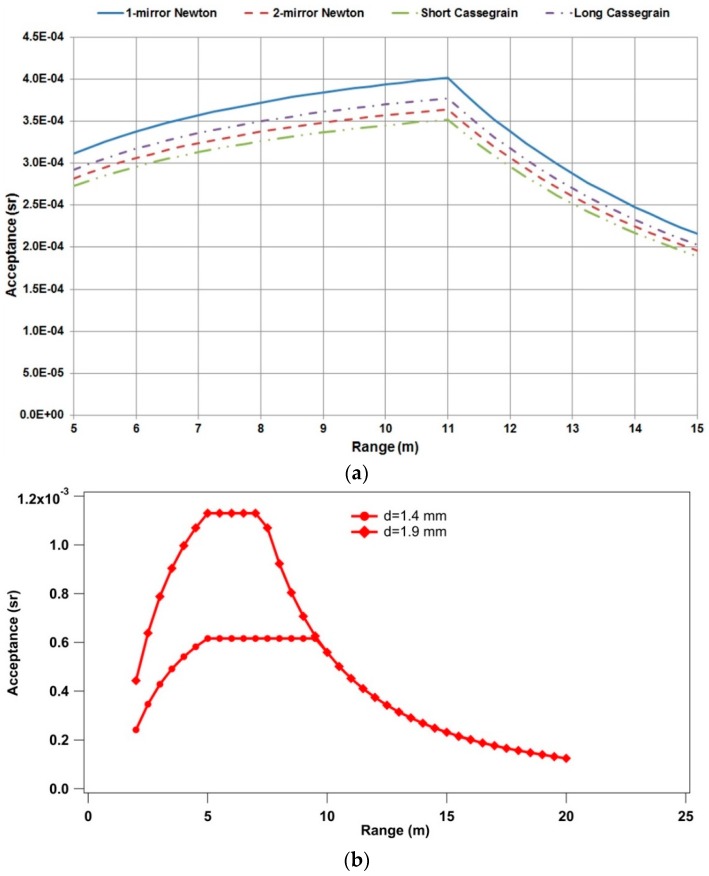
Simulated acceptance (sr) as function of the target-telescope distance for different telescope configurations (**a**); and for 2 fiber bundles with different diameters (**b**).

The coaxial geometry was chosen because it allows for measurements of samples at different distances without the need of realignment of the collimator. Moreover, the 1-mirror Newton collimator is the most suitable choice because it has the best acceptance if compared with other telescopes having the same diameter and focal length (Figure 3), and it is easier to align because it is directly focused onto the fiber bundle. The curve is the superposition of two curves: one that increases, reaching the maximum for a target distance of 11 m, *i.e.*, when the light cone coming from the parabolic mirror overlaps perfectly the collection bundle to 11 m (before 11 m some light is lost, simply because the cone footprint on the bundle surface is larger than the bundle itself); and one that decreases, due to the usual factor inversely proportional to the square of the distance (after 11 m no light is lost). Geometrical optics showed that with a telescope with a diameter of 315 mm, focal length of 1 m, and a fiber bundle with a core diameter of 200 μm, the maximum telescope acceptance of the Raman signal is obtained when the distance between the target and the mirror is 5.2–7.2 m (Figure 3) matching perfectly the requirements of the test scenario (system-target distance of about 6 m). Besides the geometry of the apparatus, the maximum Raman signal collection also depends on the telescope reflectance (98%), the filter transmittance (80%), the fiber bundle fill factor (59%) and transmittance (68%), the reflectance of the spectrometer (60%–70%) and the quantum efficiency of the detector (60%), where the weaker point in the apparatus is at the moment represented by the fiber bundle. Nevertheless, a direct telescope-spectrometer coupling is unpractical in a field experiment, leading easily to accidental misalignment and, eventually, to even higher energy losses.

**Figure 4:**
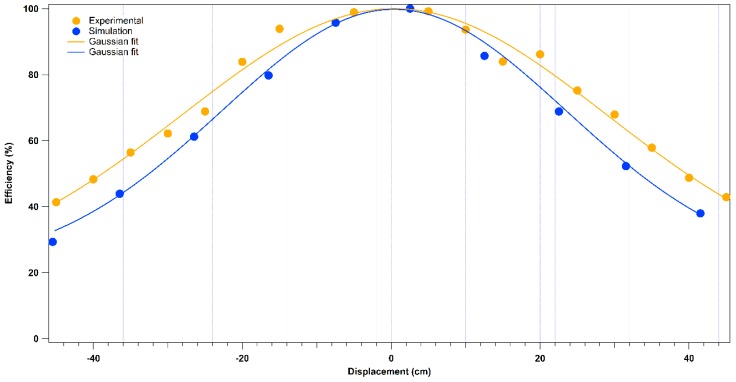
Experimental and simulated efficiency (%) in the Raman signal collection from the Teflon band at 731 cm^−1^ for displacements from the optimized target-telescope distance (cm).

After the system was aligned and optimized for the maximum Raman signal collection from Teflon at a distance of 6.4 m, various proximal measurements were performed moving the target from the optimal telescope-target distance to evaluate the effect on the collected signal. Figure 4 shows the change in the efficiency in the collection of the Raman signal from the Teflon band at 731 cm^−1^ versus the spatial offset of the target (D× [cm] = (target-collimator distance)−640 cm). The peak height was chosen as approximation of the peak area since the peak width was constant. The dots from the experimental results represent the average from 150 spectra and a step of 5 cm in distance. A Gaussian function was chosen for the fitting. For a displacement of 5 cm the signal acquired is still 98.6% of the maximum value. A 50%-decay in the Raman signal is obtained when the target is 40 cm away from the optimal distance. The experimental results are in agreement with the results obtained running the Zemax ray tracing software, thus confirming that the optics of the apparatus were modeled correctly.

### 3.2. Acquisition Software

The experimental apparatus was controlled by a software purposely designed to take care of several complementary aspects concerning the system management, including the low level control and hardware set up, the data acquisition, the data reduction and processing and finally the presentation of the results to the user.

In order to operate the sensor in public area access, the identification of substances was performed operating the apparatus in a way strictly compliant with the current rules for eye-safe operation, therefore strong limitations had been respected on the total laser energy density and on the number of laser shots. The optical receiver was highly optimized to minimize optical losses, and the thermal noise on the CCD was minimized by cooling down to −70 °C. Because of the single shot operation, low laser fluence and the fact that the light echo is revealed at 6–7 m from the target with high spectral resolution, only a low SNR is achieved, namely in the range of 1 to 10. The initial attempt to reduce the experimental data based on multivariate analysis of a broadband spectral region, did not work because of the combined effects due to low SNR and the presence of a highly variable spectral baseline, hence an original and alternative approach was chosen.

The raw data is filtered to eliminate the uncorrelated noise and to subtract the background including the fluorescence contribution, then a discrete wavelet transform of the spectrum is performed to obtain a preprocessed spectrum. The explosive discrimination, based on the classical scheme of peak recognition and the minimization of some kind of distance in a suitable space between the spectrum under test and a reference data base, was abandoned since it resulted in high number of false negatives. This was caused by a large variability of the spectral baselines due to the heterogeneous nature of the substrates. A fuzzy approach was adopted instead, giving as result the presence probability in percentage of a hazardous species (explosive).

**Figure 5:**
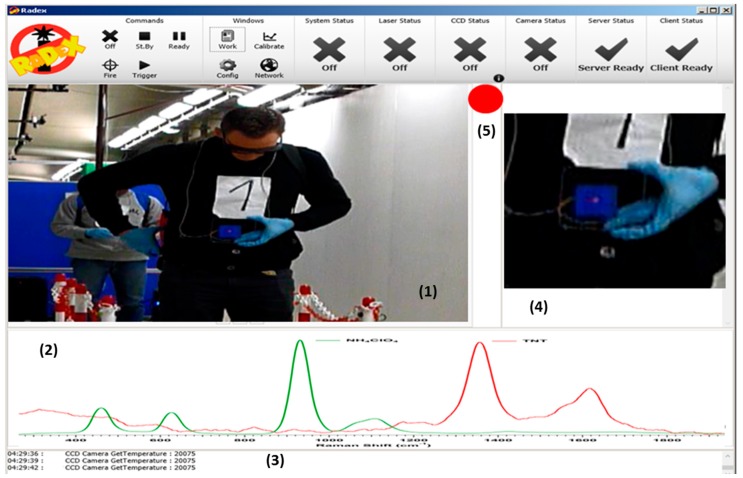
Layout of the RADEX acquisition software: streaming video centered on the point to analyze (**1**); the acquired Raman spectrum (**2**); the logging data and messages to the operator (**3**); the small field of view camera snapshot of the analyzed area (**4**); and the real time result of the analysis in form of a traffic light showing the alarm level (**5**).

The layout of the control software is captured as a snapshot and shown in Figure 5. It provides the user with the following information: streaming video centered on the point to analyze (1), the acquired Raman spectrum (2), the logging data and messages to the operator (3), the small field of view camera snapshot of the analyzed area (4) and the real time result of the analysis in form of a traffic light showing the alarm level (5).

The developed software is user friendly for the operator; only few preliminary steps are needed for the calibration and setup, moreover restricting the outcome of the analysis to the cases threat or no threat, respectively indicated with a red or green signal, facilitates the application in a real scenario where hundreds of analyses must be performed.

The program for control and acquisition is capable of sending the analysis result over a LAN in form of XML frames reporting the alarm status; for reporting purposes the control software can provide on demand the complete documentation of each probed point. According to the current status of the software development, the details stored in a permanent storage medium are: the picture documenting the sampling point, the Raman spectrum, a table with precursors or explosives searched for, the estimate of the presence for any of the explosives reported in the library. The developed software allows for a deployment of the RADEX apparatus either as a standalone configuration or as part of a more complex warning infrastructure made of several sensors.

### 3.3. Raman Spectra from Point Measurements for the Library of the Acquisition Software

**Figure 6:**
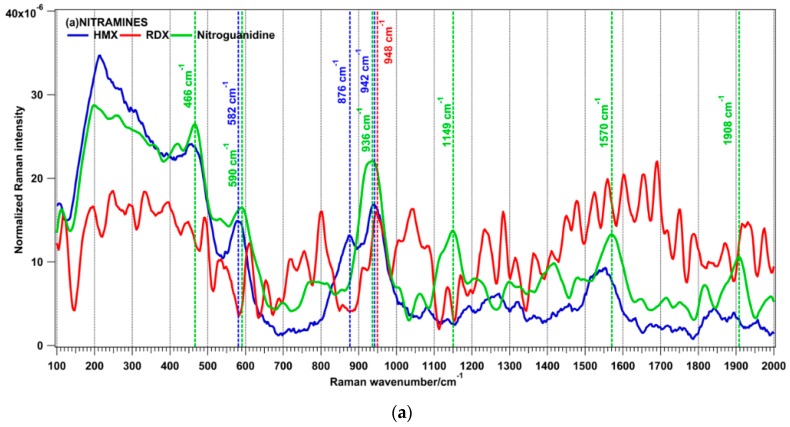

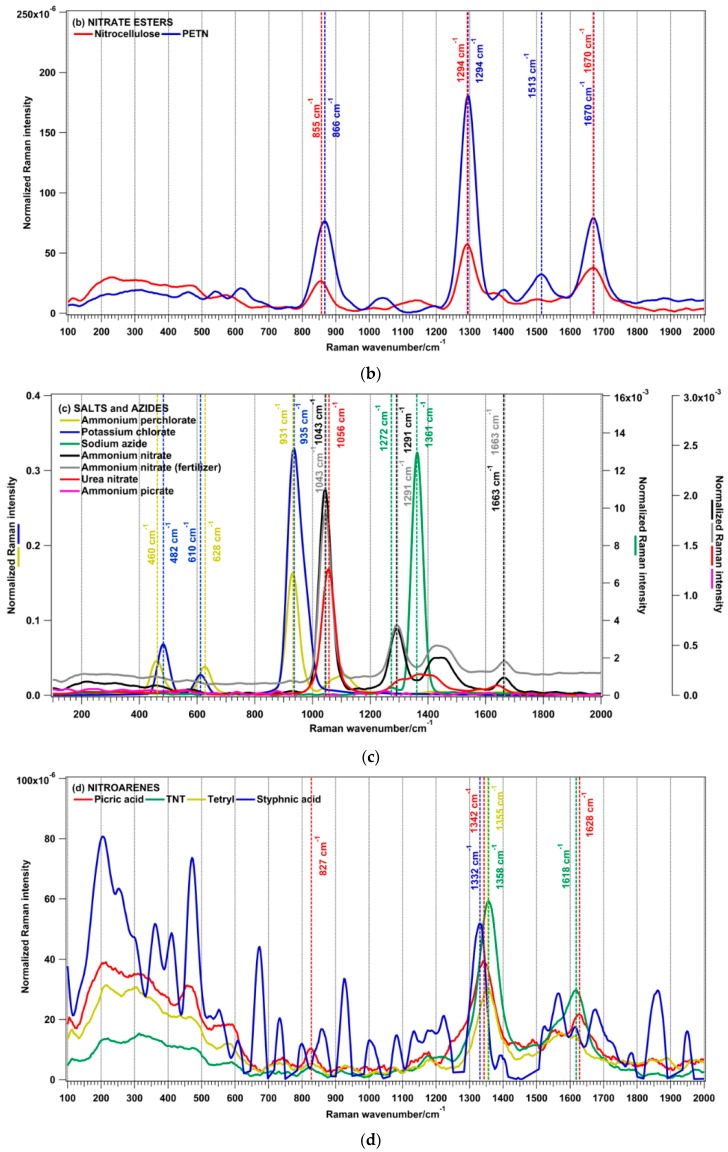

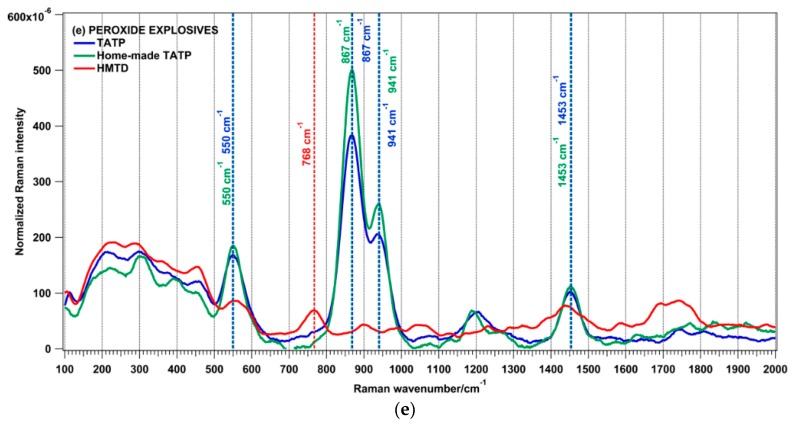
Normalized reference spectra of energetic materials: Nitramines (**a**); Nitrate esters (**b**); Salts and azides (**c**); Nitroarenes (**d**); Peroxides (**e**). Spectra acquired using the 266-nm wavelength for the excitation (pulse length = 10 ns, wavelength = 266 nm, radiant exposure = 3 mJ/cm^2^, exposure time = 0.0011 s).

Reference Raman spectra of several energetic materials (nitrate esters, nitroarenes, nitramines, peroxides, azides and oxidizer salts) were acquired to build the library of the acquisition software of RADEX. The normalized Raman spectra of the explosives in the spectral wavenumber region 100–2000 cm^−1^ are shown in Figure 6. After the background correction, the fluorescence contribution to the Raman spectrum was removed using a cubic fitting for the baseline provided by the Multipeak fitting package developed for the IGOR Pro software (Wavemetrics, Lake Oswego, OR, USA). The Savitzky-Golay smoothing was used, controlling each time the polynomial order and the number of points to compute the best smoothing filter, to remove short-term variations. The normalization of the Raman spectra, for comparison studies, was performed using Teflon as an external standard. For each analyzed sample a Raman spectrum of Teflon was acquired in the same conditions and the area of the Teflon peak at 731 cm^−1^ (stretching mode with A_1_ symmetry of CF_4_) was used for the normalization of the spectrum of the energetic compound. The area under the peak at 731 cm^−1^ is the best candidate for a scattering standard and is a more precise measure of Raman response during multiwavelength Raman measurements rather than peak height [21]. The baseline of the Teflon spectrum was initially corrected and zeroed, and the area of the Teflon band was calculated using a Gauss fit.

The explanations of the vibrational features in the Raman spectra can be found in studies already published [13,20,22,23,24,25,26,27,28,29] thus, we report here just the position of the main Raman peaks of each compound.

Nitramine molecules contain an NO_2_ group bound to a nitrogen atom (RNNO_2_). In the HMX Raman spectrum the following bands can be observed: 582 cm^−1^, 876 cm^−1^ and 942 cm^−1^. A noisy spectrum was obtained for RDX where a band at 948 cm^−1^ is observed, while for nitroguanidine the most intense band is that one at 936 cm^−1^. The acquisition of Raman spectra from RDX is very challenging for UV excitation, even with weak laser power. A Raman spectrum of RDX with few observable spectral features, obtained with a 266-nm excitation wavelength, was also observed by Gaft and Nagli [12]. Nitrate esters, *i.e.*, esters of nitric acid and alcohols, are organic molecules with the formula RONO_2_ (R = organic residue). Similar key signature bands were obtained for nitrocellulose and PETN (bands at 855–866 cm^−1^, 1294 cm^−1^, 1670 cm^−1^). Many oxidizer salts are used in explosive formulations with suitable fuels (nitrate ammonium prills mixed with fuel oil or ANFO, potassium nitrate as oxidizing agent in black powder). The most intense line in the UN spectrum was located at 1056 cm^−1^, for ammonium perchlorate at 931 cm^−1^ and for AN at 1043 cm^−1^. The main band in the spectrum of KClO_3_ was located at 935 cm^−1^. A strong line at 1361 cm^−1^ was present in the spectrum of sodium azide. Nitroarenes are nitro-substituted derivatives of aromatic hydrocarbons with the most intense peaks located at 1332–1358 cm^−1^. In the peroxide explosives the O-O bond is the source of oxygen that can produce rapid self-oxidation and explosion. The main Raman peaks associated to this chemical family are: 550 cm^−1^, 867 cm^−1^, 941 cm^−1^, 1453 cm^−1^.

**Figure 7:**
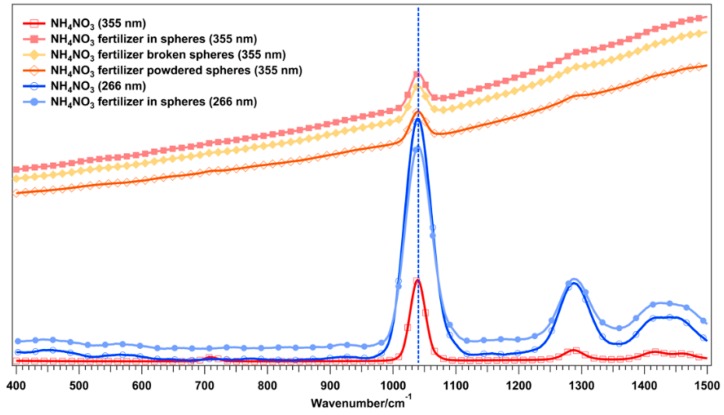
Raman spectra of ammonium nitrate and ammonium nitrate in prills (fertilizer) acquired using 266 nm and 355 nm as exciting laser.

Another advantage of using the 266-nm wavelength for the excitation instead of higher wavelengths is shown in Figure 7, where the normalized Raman spectra of AN and AN as ferlilizer are reported using the 266-and the 355-nm wavelength for the excitation (3 mJ/cm^2^ for both excitation wavelengths). The fertilizer is usually coated with an anti-caking compound to prevent sticking and clumping. Small quantities of carbonate minerals are sometimes added prior to solidifying, which eliminates the explosive properties of ammonium nitrate. At 266 nm the Raman spectrum from the prills containing AN was free of interfering fluorescence and a Raman spectrum similar to that one from pure AN was obtained, while the Raman spectrum from AN as fertilizer, acquired using a 355-nm laser pulse for the excitation, clearly shows the interference from the fluorescence even when the spheres were converted to powder.

### 3.4. Laboratory Measurements

**Figure 8:**
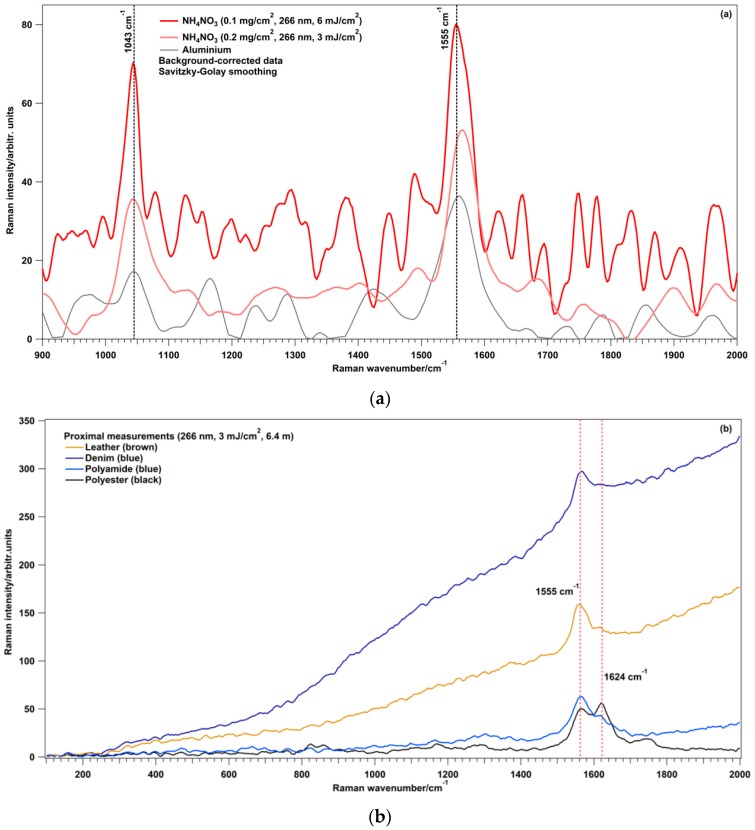

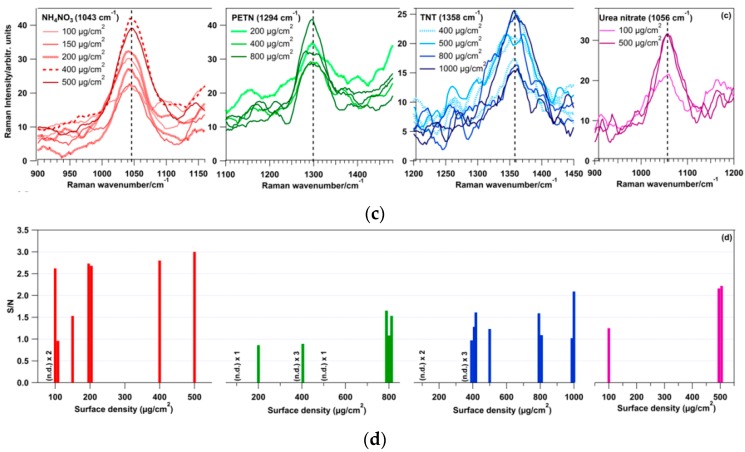
Raman spectra from aluminium and AN on aluminium at a distance of 7 m (266-nm single laser pulse at 3 and 6 mJ/cm^2^) (**a**); Four different fabrics at a distance of 6.4 m (266-nm single laser pulse, 3 mJ/cm^2^) (**b**); AN, PETN, TNT and UN at different surface densities and at a distance of 6.4 m (266-nm single laser pulse, 3 mJ/cm^2^) (**c**); and signal to noise ratios from the Raman bands reported in (**c**) (n.d. = not detected) (**d**).

Preliminary laboratory tests were performed with samples of AN on aluminium when a first version of the prototype was ready. Figure 8a shows the Raman spectra from two representative experiments of the laboratory tests. The first one was performed using one single 266-nm laser pulse with 6 mJ/cm^2^ and a sample with a surface density of 0.1 mg/cm^2^ while, the second one with 3 mJ/cm^2^ and 0.2 mg/cm^2^. The distance between the target and the telescope was 7 m. The peak at 1043 cm^−1^, assigned to the totally symmetric vibration of NO_3_ ions, was clearly observed even though low fluences (3–6 mJ/cm^2^) and surface densities (0.1–0.2 mg/cm^2^) were used. The Raman spectrum of ammonium nitrate is in agreement with previously reported spectra in literature [13]. The Raman band at 1555 cm^−1^ is from the atmospheric oxygen (another peak from N_2_ was observed at 2331 cm^−1^). The presence of the atmospheric O_2_ Raman band in the UV spectrum could mask the Raman bands from energetic materials in the same spectral region during proximal measurements of trace amounts. For instance, the asymmetric NO_2_ Raman band of the nitroaromatic explosives could be completely masked.

A measurement campaign was also performed at ICT (Pfinztal, Baden-Württemberg, Germany), where samples of energetic materials deposited on fabrics at different surface densities were used to test the RADEX apparatus [30]. The samples were prepared with PETN, TNT, AN, UN deposited on denim, leather, polyamide and polyester. Since Raman spectroscopy can be applied whenever the sample is not hidden or covered but easily reachable by the laser excitation beam, with the exception of opaque plastics, the deposited material must be optically accessible in order to favor the detection of the substance [31]. Thus, the detection of the Raman signal from samples prepared using the PNP also depends on the type of fiber used as substrate. Fibers can have different textures resulting in a wide range of mean diameter of the pores. Bigger size of the pores affects the penetration of the solution in the textile resulting in a lower surface deposition of the sample and in a higher number of particles trapped into the textile. Even if detailed studies about the penetration of the solutions through the fabrics were not performed, it seems that the pores of the denim and the leather allowed for the penetration of the solutions deposited by the PNP. Consequently, the samples with denim and leather as substrates provided signals not detected by RADEX also when the same surface densities were easily detected with other substrates. The water-proof materials, such as polyamide, were the best fabrics to use for the surface deposition of energetic materials with the PNP since the penetration of the solution through the pores could be considered negligible. The Raman spectra of the fabrics used as substrates were acquired with proximal measurements. UV excitation with 266 nm improves the detection of the Raman signal because the interference from the background luminescence is weaker and partially it is spectrally separated from the Raman region. However, fabrics can still provide interfering fluorescence even if the excitation wavelength is 266 nm (Figure 8b).

Although Raman spectroscopy of explosives on fabrics, with a 785-nm excitation wavelength, showed the presence of spectral bands arising from natural and synthetic polymers and dyed textiles [32], Figure 8b shows the absence of bands that could interfere with the identification of the explosives for wavenumbers below 1555 cm^−1^. The acquisition software was implemented to take in consideration the interference from fluorescence from the substrates and the interfering Raman peak at 1555 cm^−1^ from O_2_.

The response of the instrument in presence of AN, TNT, PETN and UN at trace levels on blue polyamide is shown in Figure 8c. The main features in the Raman spectra were the following: 1358 cm^−1^ for TNT (NO_2_ symmetric stretching coupled to CN stretching); 1043 cm^−1^ for AN (totally symmetric vibration of NO_3_ ions); 1294 cm^−1^ for PETN (NO_2_ symmetric stretching with a minor contribution from the CH bending, CH_2_ wagging, and C_5_ skeletal vibrations); 1056 cm^−1^ for UN (symmetrical stretching vibration of NO_3_). These Raman bands were chosen as most important peaks for the explosive detection with the RADEX software.

The signal to noise ratios (S/σ_y_), from the bands in Figure 8c, are reported in Figure 8d. The S/σ_y_ were calculated in the following way: (1) S is defined as the Raman peak area above background and it was obtained after the Savitzky-Golay smoothing was applied to the peak, the subtraction of the background and the fluorescence contribution to the Raman spectrum; (2) σ_y_ was obtained by subtraction of two successive spectra from the same sample, to remove the contributions of the Raman band and background and leave only the noise, and calculating the standard deviation in the peak region divided by 2^1/2^.

Even if some of the results were discordant, the RADEX apparatus was able to detect surface densities below 1000 μg/cm^2^ at a distance of 6.4 meters using only one laser pulse with 3 mJ/cm^2^. A possible explanation for some of the discordant results is the fact that during the campaign the deposition method of different energetic materials with the PNP was in an optimization stage (for instance choice of the best solvent, optimum solution concentrations, etc) for the upcoming trials in Paris.

AN was the substance that provided the highest S/σ_y_ ratios, in agreement with its Raman cross section, and together with UN were the only two substances to provide detectable signals also when the surface density was 100 μg/cm^2^. The lower detectable surface densities of PETN and TNT on polyamide were 200 and 400 μg/cm^2^, respectively.

**Figure 9:**
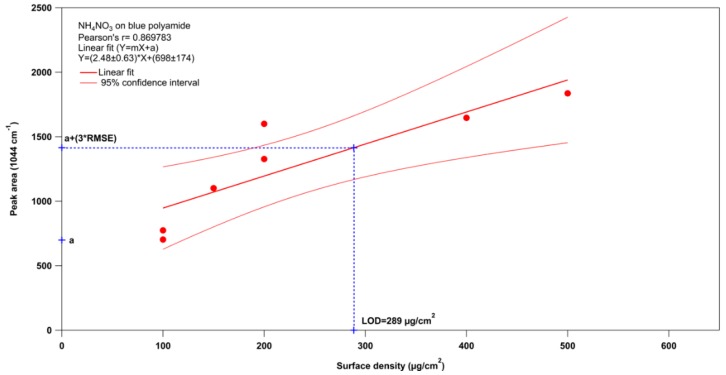
Limit of detection for AN deposited on blue polyamide.

An estimated limit of detection (LOD) was calculated for AN using the data collected at ICT and some measurements performed at ENEA. The method used to estimate the LOD is the one that involves the generation of a calibration curve and the calculation of the root mean square error (RMSE). This method can be applied when a linear relation between the detector response and the quantity of analyte is observed. The regression analysis of the calibration curve is performed to obtain slope and intercept. Then, the RMSE is calculated and the LOD is obtained with the formula [(3 × RMSE)/m], where m is the slope of the linear fit. The LOD calculated is 289 μg/cm^2^ as shown in Figure 9. This is just a first indication of the LOD of RADEX during proximal measurements of AN on polyamide, because more measurements are necessary together with more information about particle dimensions and penetration depth of the laser. The maximum penetration depth of UV-laser light into a sample is typically of the order of a tenth of a micron to microns and this aspect should be taken into account for an accurate evaluation of the detection limits of the instrument. For thin samples of few nm the absorption can be considered negligible at various wavelengths in the UV range and the relative Raman signal closely follows the cross section. When the samples have a thickness of few microns, absorption starts to become an important effect going in the UV range, and the Raman signal does not increase as the cross sections would suggest [33]. Detailed studies on the dimensions of the deposited particles on the substrates by the PNP, and the penetration depth of the laser, were not performed to better define the real detection capability of the apparatus.

### 3.5. Field Measurements

**Figure 10:**
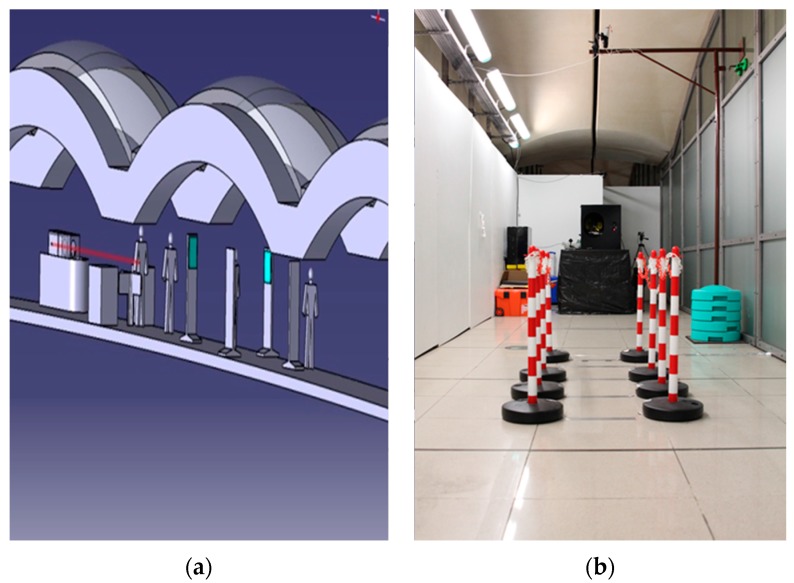
RADEX, and gate with sensors, 3D-model (**a**) and set-up (**b**) during the trials in the Bibliothèque François Mitterrand (BFM) metro station. Optical sensors were used to simulate a gate and to synchronize the laser emission with the transit of a passenger. The RADEX system sent an eye-safe laser shot to the suspect person passing through the gate.

During the BCT trials, the RADEX prototype was placed in the footbridge of the Bibliothèque François Mitterrand (BFM) metro station as shown in Figure 10 looking into the direction opposite to the passenger flow with the laser placed at the specified height of 1.15 m from the ground.

Two other sensors, and a video tracking system, where placed in the footbridge together with RADEX. The data from each apparatus were collected and merged by a data merging and alert system (DAMAS; CEA, Paris, France).

A pseudo-gate was installed in front of the RADEX instrument. Optical sensors were used to simulate a gate at the end of the delimited corridor and to synchronize the laser emission with the transit of a passenger. The corridor was delimited in order to guide the passengers in direction of the instrument and the sensors were placed at a distance of 6.4 m from the telescope. The optics of RADEX were aligned after the installation, and the laser fluence was measured with an energy meter and it was set to the nominal value of 3 mJ/cm^2^. The laser fluence was certified by the Laboratoire National de Métrologie et d’Essais (Paris, France). Calibration of the spectral interval was performed using the Teflon Raman peaks as references each day before the measurements.

Each time DAMAS sent a “shot” command to RADEX, after the identification of a suspect person by the other instruments, the RADEX system sent automatically an eye-safe laser shot to the suspect person passing through the gate. The acquired Raman signal was then analyzed in real time and the response, together with all the stored information, sent to DAMAS for each event.

Four different types of fabrics (blue denim, red polypropylene, blue polyamide and brown leather) were supposed to be used as substrates during the trials, but at the end only denim and polyamide didn’t interfere with the Raman measurements. Samples with leather as substrate didn’t provide detectable signal confirming the findings during the campaign at ICT, whereas polypropylene was associated with a high fluorescence signal responsible of false alarms. Thus, we report here only the results of the measurements with denim (fingerprint samples) and polyamide (trace analysis) as substrates. The tested energetic materials were: AN, UN, TNT and PETN.

The first tests were performed only with fabrics (denim, polyamide and leather) to evaluate the percentages of false positives. After the first set of non-threat measurements, the false alarm events were 18 out of 31. False alarms where caused by the interference of the atmospheric O_2_-Raman band at 1555 cm^−1^. A narrow rejection region was already imposed (from 1520 to 1600 cm^−1^) to take the O_2_ peak into account and to avoid the exclusion of other bands from energetic materials, but the tails of the strong O_2_ band often exceed the threshold at the edges of this rejected region leading to numerous false attributions.

This software problem was solved after the first session of blank experiments by extending the blind zone from 1490 to 1610 cm^−1^. This procedure was effective also considering that the Raman peak from TNT (1510 cm^−1^) and PETN (1614 cm^−1^) in this region are weak and were not expected to be easily revealed at the experimental conditions. Widening the blind zone makes the system less prone to this error for the rest of the trials. During the second session of blank measurements, 115 non-threat tests were performed with a 0% false alarm rate. Also the tests from the first session were reprocessed and the number of false positives obtained were only 1 for a leather substrate.

**Figure 11:**
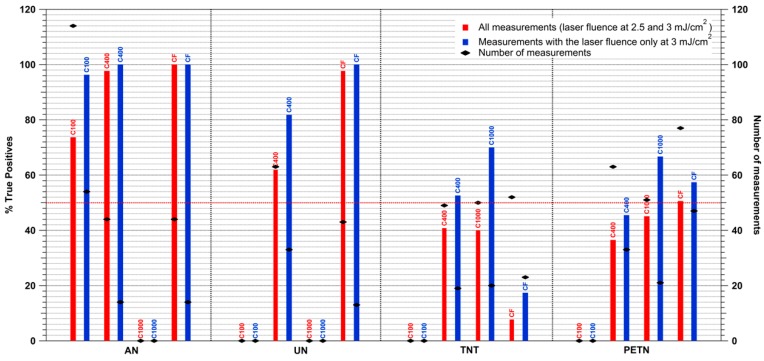
Percentages of true positives for AN, UN, TNT and PETN from al measurements with laser fluence at 2.5–3 mJ/cm^2^ and for all measurements with laser fluence at only 3 mJ/cm^2^.

The total number of tests performed with fingerprint and trace samples were 216 and 434, respectively. During the first set of measurements, 119 fingerprint tests and 240 trace tests, the laser fluence was on average around 2.5 mJ/cm^2^ due to technical problems with the laser. In Figure 11 are reported the percentages of true positives, number of alarm events over the total number of samples analyzed, and the total number of measurements for TNT, PETN, AN and UN with the following surface densities: 100 μg/cm^2^ (C100), 400 μg/cm^2^ (C400), 1000 μg/cm^2^ (C1000), fingerprints (CF). The percentages of true positives obtained considering all the measurements performed (fluence = 2.5–3 mJ/cm^2^) are shown by the red bars in Figure 11. For the fingerprint samples the percentages of true positives for AN and UN were 100% and 98%, respectively while, it was 8% for TNT and 51% for PETN. For the trace analysis the percentages of true positives were 74% for AN-C100, 98% for AN-C400, 62% for UN-C400, 41% for TNT-C400, 40% for TNT-1000, 37% for PETN-C400, 45% for PETN-C1000. Promising results were obtained for AN and UN also with the experiments performed with the laser fluence of only 2.5 mJ/cm^2^.Taking in consideration only the data acquired with a laser fluence of 3 mJ/cm^2^ (97 fingerprint samples, 194 trace samples) higher percentages of true positives were obtained (blue bars in Figure 11): 100% for AN-CF and UN-CF, 17% for TNT-CF, 57% for PETN-CF, 96% for AN-C100, 100% for AN-C400, 82% for UN-C400, 53% for TNT-C400, 70% for TNT-1000, 46% for PETN-C400 and 67% for PETN-C1000.

## 4. Conclusions

The first Raman LIDAR instrumentation, for civil applications, was developed for the proximal detection of trace explosives on fabrics (cloths or backpacks) taking in consideration the constraint of the maximum permissible laser exposure of the human cornea and skin. To our knowledge, this is the first Raman apparatus to be tested with real people as target and in conditions as close as possible to a realistic scenario in a metro station in Paris during 3-week trials. Proximal measurements were performed in presence of AN, TNT, PETN and UN deposited on different fabrics. Using polyamide as substrate the RADEX apparatus was able to detect surface densities of AN, TNT, PETN and UN in the range 100–1000 μg/cm^2^ with the samples at a distance of 6.4 m and using a single laser pulse of only 3 mJ/cm^2^.

AN and UN provided the highest percentages of true positives (>82% for surface densities of 100–400 μg/cm^2^ and fingerprints) followed by TNT and PETN (17%–70% for surface densities of 400–1000 μg/cm^2^ and fingerprints). The limit of detection calculated in first approximation for ammonium nitrate on polyamide was 289 μg/cm^2^.

An user-friendly acquisition software was developed for an on-line analysis of the data and to provide a quick response to an operator (alarm or clear). This software allows for a deployment of the RADEX apparatus either stand alone or as part of a more sophisticated warning system architecture made of several sensors. RADEX was a robust apparatus because the challenging environmental conditions of the metro station (*i.e.*, dust, temperature, electromagnetic fields, humidity, *etc.*) didn’t interfere with the instrument.

Polypropylene could not be used as substrate because it provided false alarms caused by the interference from its fluorescence in the Raman spectra. The software will be improved to better take in consideration the interfering fluorescence produced by substrates and also potential technical improvements, such as the utilization of a picosecond laser and an intensified CCD camera to gate in shorter time close to the excitation, will be taken in consideration for the new version of the prototype.

The area investigated by the instrument was only 1 cm^2^, which makes the percentage of hitting an area contaminated with explosives really low. A newer version of the apparatus will be developed with a scanning system to face the limitation of this prototype.

The current state of development of the RADEX apparatus, with respect to the Technology Readiness Level (U.S. Department of Defense), is between 6 and 7 since it was tested in a real metro station simulating a real operational environment.
